# Supplementary material for: Intra-group differences in skin tone influence evaluative and perceptual face processing
Source: PLoS One. 2024 Jan 2;19(1):e0296172. doi: 10.1371/journal.pone.0296172 (PMC10760692; doi:10.1371/journal.pone.0296172)
Supplement: S1 Table — (DOCX) [file pone.0296172.s001.docx]

# Supporting Information

# S1: Effects of stimulus characteristics on attractiveness ratings

Table S1. Exploratory 4-way ANOVA explaining attractiveness as a function of stimulus characteristics

| *Effects/Interactions* | *Summary* |
| --- | --- |
| **Tone** | **F (1, 304) = 31.9; p = 0.001; eta2_p = 0.095** |
| **Sex** | **F (1, 304) = 160.8; p = 0.001; eta2_p = 0.346** |
| **Attractive** | **F (1, 304) = 1156.67; p = 0.001; eta2_p = 0.792** |
| **Ethnicity** | **F (3, 912) = 10.63; p = 0.001; eta2_p = 0.034** |
| **Tone:Sex** | **F (1, 304) = 75.45; p = 0.001; eta2_p = 0.199** |
| Tone:Attractive | F (1, 304) = 0.14; p = 0.706; eta2_p = 0 |
| Sex:Attractive | F (1, 304) = 6; p = 0.015; eta2_p = 0.019 |
| **Tone:Ethnicity** | **F (3, 912) = 106.59; p = 0.001; eta2_p = 0.26** |
| **Sex:Ethnicity** | **F (3, 912) = 4.15; p = 0.006; eta2_p = 0.013** |
| **Attractive:Ethnicity** | **F (3, 912) = 75.17; p = 0.001; eta2_p = 0.198** |
| Tone:Sex:Attractive | F (1, 304) = 1.21; p = 0.272; eta2_p = 0.004 |
| **Tone:Sex:Ethnicity** | **F (3, 912) = 16.99; p = 0.001; eta2_p = 0.053** |
| **Tone:Attractive:Ethnicity** | **F (3, 912) = 84.3; p = 0.001; eta2_p = 0.217** |
| **Sex:Attractive:Ethnicity** | **F (3, 912) = 21.56; p = 0.001; eta2_p = 0.066** |
| **Tone:Sex:Attractive:Ethnicity** | **F (3, 912) = 23.72; p = 0.001; eta2_p = 0.072** |

* All statistically significant effects/interactions marked in bold.

Across studies, our stimulus sample was varied along target ethnicity (4), sex (2), attractiveness (2) and skin tone (2). A 4 x 2 x 2 x 2 (*k* = 32) repeated-measures analysis of variance (rANOVA) explored whether mean-centered ratings could be statistically explained. ANOVAs were run on median (not raw) estimates across factor levels to achieve a balanced design. Levene’s test indicated variances were not homogenous across measurements (*p* = 0). Shapiro tests indicated mean-centered ratings were normally distributed across 12 out of 32 measurements. Sphericity violations were addressed using Greenhouse-Geisser corrections. 3/4 three-way and 5/6 two-way interactions reached significance (all *p*’s $\leq$ .015; all $\eta_{p}^{2}>$’s .013). Main effects were significant for all factors *viz* skin tone, target sex, ethnicity and attractiveness levels (all *p*’s $\leq$ .001; all $\eta_{p}^{2}>$’s .034; see Table 1).

Following the significant 4-way interaction detected above, 16 Holm-adjusted post-hoc contrasts between lighter and darker variants nested by target sex, ethnicity and attractiveness level found reliable evidence for lighter Black *HAF*, *t*(588.3) = -6.99; *p* = .001; *g*[95%] = -0.29 [-0.37, -0.21]; Asian *HAM*, *t*(593.2) = -13.35; *p* = .001; *g*[95%] = -0.55 [-0.63, -0.46]; Black *HAM*, *t*(598.6) = -8.27; *p* = .001; *g*[95%] = -0.34 [-0.42, -0.26]; Asian *LAM*, *t*(607.9) = -3.84; *p* = .001; *g*[95%] = -0.16 [-0.24, -0.08]; Black *LAM*, *t*(601.6) = -3.34; *p* = .001; *g*[95%] = -0.14 [-0.22, -0.06]; and Latinx *LAM*, *t*(592.8) = -4.47; *p* = .001; *g*[95%] = -0.18 [-0.26, -0.1], being evaluated as more attractive than their darker counterparts. Conversely, darker Latinx *HAF*, *t*(608) = 9.4; *p* = .001; *g*[95%] = 0.38 [0.3, 0.46]; Latinx *HAM*, *t*(601.3) = 8.72; *p* = .001; *g*[95%] = 0.36 [0.27, 0.44]; and White *LAF*, *t*(605.8) = 2.66; *p* = 0.008; *g*[95%] = 0.11 [0.03, 0.19], were evaluated as more attractive than their lighter counterparts.

We next explored for directionality across all main effects reported. A post-hoc two-sample Welch’s test confirmed mean attractiveness for female targets (2.97 ms) was significantly greater, $t_{Welch}\left( 9748.2 \right)=15.51;p=.001;g_{Hedge}\left[ 95CI \right]=-0.31\left[ -0.35,-0.27 \right]$ than for male targets (2.63). A second Welch’s test confirmed lighter targets (2.84) were evaluated as significantly more attractive, $t_{Welch}\left( 9757.5 \right)=3.34;p=.001;g_{Hedge}\left[ 95CI \right]=-0.07\left[ -0.11,-0.03 \right]$, than darker targets (2.76). A third Welch’s test confirmed High-Attractive faces (3.45) were evaluated as more attractive, $t_{Welch}\left( 9106.4 \right)=75.27;p=.001;g_{Hedge}\left[ 95CI \right]=1.52\left[ 1.68,1.68 \right]$, compared to Low-Attractive faces (2.15). Finally, a one-way ANOVA comparing mean attractiveness ratings between Asian (2.73), Black (2.80), White (2.80) and Latinx (2.86) targets reached significance, *F*(3, 9756) = 5.97, *p* < .001, $\eta_{p}^{2}$ < .01. Tukey’s HSD tests indicated only the mean difference between Asian and Latinx target attractiveness to reach statistical significance (*p* = 1e-04).
