# Supplementary material for: Intra-group differences in skin tone influence evaluative and perceptual face processing
Source: PLoS One. 2024 Jan 2;19(1):e0296172. doi: 10.1371/journal.pone.0296172 (PMC10760692; doi:10.1371/journal.pone.0296172)
Supplement: S2 Table — (DOCX) [file pone.0296172.s002.docx]

# Supporting Information

# S2: Effects of stimulus characteristics and facial orientation on breaking times

Table S2. Exploratory 4-way ANOVA explaining median breaking times as a function of stimulus characteristics

| *Effects/Interactions* | *Summary* |
| --- | --- |
| Face | F (1, 49) = 0.07; p = 0.789; eta2_p = 0.001 |
| **Tone** | **F (1, 49) = 25.55; p = 0.001; eta2_p = 0.343** |
| **Sex** | **F (1, 49) = 70.69; p = 0.001; eta2_p = 0.591** |
| Attract | F (1, 49) = 3.6; p = 0.064; eta2_p = 0.068 |
| **Ethnicity** | **F (3, 147) = 15.28; p = 0.001; eta2_p = 0.238** |
| Face x Tone | F (1, 49) = 0.5; p = 0.484; eta2_p = 0.01 |
| Face x Sex | F (1, 49) = 0.36; p = 0.55; eta2_p = 0.007 |
| Face x Attract | F (1, 49) = 0.04; p = 0.849; eta2_p = 0.001 |
| Face x Ethnicity | F (3, 147) = 1.26; p = 0.29; eta2_p = 0.025 |
| Tone x Sex | F (1, 49) = 0.31; p = 0.579; eta2_p = 0.006 |
| Tone x Attract | F (1, 49) = 2.4; p = 0.128; eta2_p = 0.047 |
| Sex x Attract | F (1, 49) = 0; p = 0.979; eta2_p = 0 |
| Tone x Ethnicity | F (3, 147) = 2.12; p = 0.1; eta2_p = 0.041 |
| **Sex x Ethnicity** | **F (3, 147) = 2.66; p = 0.05; eta2_p = 0.051** |
| Attract x Ethnicity | F (3, 147) = 2.01; p = 0.115; eta2_p = 0.039 |
| **Face x Tone x Sex** | **F (1, 49) = 14.12; p = 0.001; eta2_p = 0.224** |
| Face x Tone x Attract | F (1, 49) = 0.13; p = 0.723; eta2_p = 0.003 |
| Face x Sex x Attract | F (1, 49) = 0.33; p = 0.569; eta2_p = 0.007 |
| Face x Tone x Ethnicity | F (3, 147) = 0.23; p = 0.874; eta2_p = 0.005 |
| Face x Sex x Ethnicity | F (3, 147) = 0.78; p = 0.506; eta2_p = 0.016 |
| Face x Attract x Ethnicity | F (3, 147) = 0.12; p = 0.95; eta2_p = 0.002 |
| **Tone x Sex x Attract** | **F (1, 49) = 9.19; p = 0.004; eta2_p = 0.158** |
| Tone x Sex x Ethnicity | F (3, 147) = 0.15; p = 0.928; eta2_p = 0.003 |
| **Tone x Attract x Ethnicity** | **F (3, 147) = 6.72; p = 0.001; eta2_p = 0.121** |
| Sex x Attract x Ethnicity | F (3, 147) = 3.77; p = 0.012; eta2_p = 0.071 |
| Face x Tone x Sex x Attract | F (1, 49) = 0; p = 0.999; eta2_p = 0 |
| Face x Tone x Sex x Ethnicity | F (3, 147) = 0.32; p = 0.809; eta2_p = 0.007 |
| Face x Tone x Attract x Ethnicity | F (3, 147) = 0.5; p = 0.683; eta2_p = 0.01 |
| Face x Sex x Attract x Ethnicity | F (3, 147) = 0.46; p = 0.712; eta2_p = 0.009 |
| **Tone x Sex x Attract x Ethnicity** | **F (3, 147) = 8.72; p = 0.001; eta2_p = 0.151** |
| Face x Tone x Sex x Attract x Ethnicity | F (3, 147) = 0.62; p = 0.603; eta2_p = 0.012 |

A 4 x 2 x 2 x 2 x 2 mixed ANOVA explored whether stimulus characteristics and face orientation significantly explained variances in median breaking times. Levene’s test indicated variances were homogenous across measurements (*p* = 0.68). Shapiro tests across breaking time distributions indicated the assumption of normality had been violated for 19 out of 64 measurements. The five-way interaction was not significant. A significant four-way interaction explained variance in median breaking times, specifically for the Tone:Sex:Attract:Ethnicity interaction term, $F_{\left( 3,147 \right)}=8.72;p=0.001;\eta_{p}^{2}=$ 0.151. 4/10 three-way interactions (all *p*’s $\leq$ .013; all $\eta_{p}^{2}>$’s .07) and a single two-way interaction between target sex and ethnicity reached significance (*p* = .05, $\eta_{p}^{2}=.238$; see Table 2. Main effects were significant for skin tone, $F_{\left( 1,49 \right)}=25.55;p=0.001;\eta_{p}^{2}=$ 0.343, target sex, $F_{\left( 1,49 \right)}=70.69;p=0.001;\eta_{p}^{2}=$ 0.591, and target ethnicity, $F_{\left( 3,147 \right)}=15.28;p=0.001;\eta_{p}^{2}=$ 0.238, respectively. As before, the significant four-way interaction warranted 16 Holm-adjusted contrasts between breaking times recorded for lighter and darker variants across nested factor levels. Breaking times were significantly faster for lighter Asian *HAM*, *t*(113.5) = 2.42; *p* = 0.017; *g*[95%] = 0.23 [0.04, 0.41], lighter White *HAM*, *t*(114.6) = 2.94; *p* = 0.004; *g*[95%] = 0.27 [0.09, 0.46], and lighter Black *LAM*, 4, relative to their darker counterparts.

We additionally explored for directionality across the main effects reported above. A post-hoc two-sample Welch’s test confirmed mean breaking times for female targets (5812 ms) were significantly shorter, $t_{Welch}\left( 1848.8 \right)=4.97;p=.001;g_{Hedge}\left[ 95CI \right]=0.23\left[ 0.14,0.32 \right]$, relative to breaking times for male targets (6107). A second Welch’s test confirmed lighter targets (5878) broke suppression significantly faster, $t_{Welch}\left( 1853.1 \right)=2.75;p=0.006;g_{Hedge}\left[ 95CI \right]=0.13\left[ 0.04,0.22 \right]$, than darker targets (6042). Finally, a one-way ANOVA comparing mean breaking times between Asian (5908), Black (6120), White (5913) and Latinx (5896) targets reached significance, . Tukey’s HSD tests indicated only the difference between Black and Latinx target mean breaking times to be statistically significant (*p* = 0.04).
